# Epidemiology of pediatric trauma in the Kingdom of Bahrain: a national pediatric trauma registry pilot study

**DOI:** 10.1186/s40621-021-00336-8

**Published:** 2021-07-05

**Authors:** Jay C. Liu, Aieshah A. Ismael, Ayesha Zaidi, Ban W. Sha’ban, Shaikha Ebrahim Almutawa, Asad Amin Chatha, Feras H. Abuzeyad, Ruqaya Isa Jaafar, Salah Ali Alghanem, Ghada Al Qassim, Nitya Kumar, Martin Corbally

**Affiliations:** 1 Royal College of Surgeons in Ireland - Bahrain, Busaiteen, Bahrain; 2 Mohammed Bin Khalifa Bin Salman Al Khalifa Specialist Cardiac Center, Awali, Bahrain; 3 Salmaniya Medical Complex, MOH, Manama, Bahrain; 4 Emergency Department, Busaiteen, Bahrain; 5 Bahrain Defence Force - Royal Medical Services, Riffa, Bahrain; 6 King Hamad University Hospital, Busaiteen, Bahrain

**Keywords:** Trauma registry, pediatric trauma, global health, trauma epidemiology, Bahrain, motor vehicle collision, seatbelt, drowning, surgery admission

## Abstract

**Background:**

A pediatric trauma registry for the Kingdom of Bahrain would be a novel public health tool for the Bahraini health system. The aim of this study was to explore the epidemiology of pediatric trauma at the national level by describing the distribution of pediatric injury in the Kingdom, and quantifying the burden of injury shouldered by the study population.

**Methods:**

This multicenter observational cross-sectional study was conducted in Bahrain using data from the Pediatric Trauma Registry (PTR), which was a short-term paper-based prospective trauma registry that collected data over a three-month period in 2018. PTR was based in the pediatric emergency departments (ED) of the three national referral hospitals in the Kingdom. By simultaneously collecting data from all three trauma hospitals in the country, it was assumed that during the data collection period all major pediatric trauma patients in the country would be captured by the study, and that the data collected would provide national estimates of trauma. Inclusion criteria for the study was any individual under the age of 14, that arrived at the ED seeking care for intentional and unintentional injuries.

**Results:**

A total of 1328 patients were included in the study. Sixty-nine percent of patients were treated and discharged from the ED, 30.5% were admitted to the hospital, admitted for surgery, or seen by a specialist, and 0.5% were declared deceased. The percentage of patients documented as unrestrained during Motor Vehicle Collisions (MVC) was 92.3%, and amongst those involved in MVC, 12% were ejected from the cabin of the vehicle.

**Conclusions:**

There are significant implications that this study holds for policy implementation and practice surrounding injury prevention in the Kingdom of Bahrain. Low seatbelt utilization and the high proportion of ejection amongst MVC victims warrant immediate public health policy implementation, including enforcement of seat belt laws, strengthening of the traffic court system, and awareness campaigns for MVC prevention. Additionally, pediatric drowning prevention programs centered on constant adult supervision, pool isolation fencing, personal flotation devices, and swimming education should be created to address the mortality attributable to drowning in this study.

## Background

Amongst children globally, trauma is a major cause of mortality and morbidity (Haagsma et al., 2016; Ameratunga & Peden, 2009). Trauma registries are able to provide accurate prospective trauma epidemiology data which can empower hospital-based trauma care systems to improve their services and enable governmental authorities to pass effective injury prevention legislation through evidence-based decision making (Moore & Clark, 2008). A pediatric trauma registry for the Kingdom of Bahrain would be a novel public health tool for the population and would strengthen the Bahraini health system by allowing it to join systems in the region, such as Saudi Arabia, Qatar, and Iran, who have implemented surveillance systems.

Data from the Global Burden of Diseases, Injuries and Risk Factors Study 2010 (Lozano et al., 2012) indicates that high income countries in the Arab world, such as Bahrain, have experienced a reduction in mortality and morbidity of preventable and infectious diseases over the previous two decades.

However, in the last two years, public health researchers and officials in Bahrain studying motor vehicle collisions (MVCs) have specifically requested for action to be taken in the surveillance of pediatric injuries from MVCs (Awadhalla et al., 2016). Current literature demonstrates that MVCs persist as a leading cause of death and disability adjusted life years (DALYs) (Mokdad et al., 2014; Asim et al., 2014).

In 2013, Bahrainis under the age of 25 were found to be 3.5 times more likely to perish in a MVC compared to the general population. Additionally, it was found that young Bahraini males were 6 times more likely to die in a MVC than young Bahraini females (Hamadeh & Ali, 2013). At the time, a call for the implementation of a trauma surveillance system in the country was made, to further explore these trends. In addition to the evolving trends in MVCs, there is also evidence that suggests the incidence of physical child abuse in Bahrain may be rising (Al-Mahroos & Al-Amer, 2012). A National Bahraini pediatric trauma registry would be invaluable in identifying at-risk groups within the pediatric population and illuminating areas where implementing intervention programs and policy changes can be most effective.

Evidence from countries with established permanent trauma registries illustrates that performing multi-pronged trauma prevention programs to reduce pediatric deaths from trauma is not only possible, but also very reproducible (Harvey et al., 2009). A trauma registry study in Qatar, a neighboring country with comparable epidemiological trends, found that head injuries, long bone injuries, and polytrauma were the most common pediatric injuries, while also finding evidence of seasonality in pediatric trauma incidence (Alyafei et al., 2015). Overall the knowledge deficit surrounding the epidemiology of trauma in the Arab World leaves policy makers unaware of the extent to which preventable trauma burdens the society (Asim et al., 2014).

The success of the registry in Qatar, as well as the trauma surveillance system in the neighboring Kingdom of Saudi Arabia, suggests that initiation of such a registry in Bahrain is possible.

Ideally this type of trauma registry would be a permanent national surveillance tool that constantly produces prospective data dynamically. This study established a short-term pilot

registry to produce an initial data set to analyze, while also attempting to uncovering the pragmatic obstacles that may arise with registry implementation.

Additionally, recent developments in emergency medicine, graduate medical education, and trauma care in Bahrain’s major governmental hospitals make installation of a prospective data collection system perfectly poised to guide subsequent advancements in the trauma system (Abuzeyad et al., 2018; Nwomeh et al., 2006).

### Objectives

The objectives of this study centered on describing the current epidemiology of pediatric injury in the Kingdom of Bahrain by exploring the distribution of pediatric trauma in tertiary hospitals in the kingdom, and quantifying the burden of injury shouldered by the study population.

## Methods

### Study design

This multicenter observational cross-sectional study was conducted in the Kingdom of Bahrain using data from the Pediatric Trauma Registry (PTR), which was a short-term paper-based prospective trauma registry based in the pediatric emergency departments (ED) of the three national referral hospitals in the Kingdom.

Prior to initiation of the registry the research team met with hospital administrators at each of the participating institutions to discuss the details of the study. Each of the collaborating hospitals had a delegated research coordinator to oversee the data collection process, to ensure production of high-quality data, and to troubleshoot and document any obstacles that arose during data collection. Hospital administrators and research coordinators informally reported pragmatic obstacles encountered to initiation and implementation of the trauma registry at their respective hospital EDs to the research team. Each institution had department wide training of all emergency physicians so that all staff were familiar with the study, and knowledgeable about when and how to complete the paper registry forms. Registry forms were exclusively completed in English by the emergency physician evaluating the patient.

In Bahrain, only patients under the age of 14 are treated in the pediatric ED. This standardized threshold age guided decision making during the study design period, when local healthcare administrators expressed concern about the pragmatic obstacles that would be encountered if the inclusion criteria were to encompass pediatric patients up to the age of 18.

### Study setting and population

Bahrain is an island nation situated in the Persian Gulf, off the coast of Saudi Arabia. It is a member state of the Gulf Cooperative Council (GCC), and is a high-income country based on GDP per capita. As of 2014 the estimated population of Bahrain was 1.316 million, with 25.9% of the population being under the age of 19, and 54.4% of the population being non-Bahraini (General Directorate of Statistics, 2016). The health system in Bahrain is rapidly developing, and medical records are in the process of transitioning from paper to electronic medical record keeping. The study population consisted of pediatric trauma patients who presented to the pediatric ED of the three major trauma hospitals in Bahrain: Salmaniya Medical Complex (SMC), King Hamad University Hospital (KHUH), and Bahrain Defense Force Hospital (BDF).

Inclusion criteria for the study was any patient under the age of 14 that arrived at the ED seeking care for injuries sustained from trauma. Exclusion criteria was any patient with an uncertain description of injury, such as those with soft tissue injuries suggestive of trauma without an identifiable description of injury. Some patients presented to the ED for medical problems with coincidental minor injuries, and these injuries alone would not have prompted an ED visit thus including them in the sample would be inappropriate.

SMC is the largest health care facility in the kingdom with an ED bed capacity of 85. BDF is the second largest hospital in the country, and its ED has 31 beds. KHUH’s ED has a total of 45 beds. All three hospitals are fully equipped tertiary care trauma centers with modern facilities capable of handling any major pediatric trauma case and rendering surgical critical care. BDF hospital houses the designated burn unit for the country.

By simultaneously collecting data from all three trauma hospitals in the country, it was assumed that during the data collection period all major pediatric trauma patients in the country would be captured by the study, and that the data collected would provide national estimates of trauma. Additionally, private tertiary hospitals in Bahrain do not accept major trauma patients, and all patients presenting to a private hospital requiring tertiary trauma care are transported to one of the three designate trauma centers, guaranteeing that all major trauma patients presenting to private hospitals were captured in this study design. Minor injuries treated in local clinics or private hospitals were not captured by this study design. Traumatic prehospital pediatric mortality was also monitored by collaborators in the Emergency Medical Services (EMS) system, and no such events were reported in Bahrain during the study period.

### Variables and information collected

The PTR collected information on patient demographics, description of the injury, location where the injury occurred, ED interventions, and disposition of all pediatric trauma patients presenting to the pediatric ED during the study period. The data collection tool incorporated components from trauma registry forms used previously by Duron et al. in Peru, the Pan-American Trauma Society, and the World Health Organization, while also being adapted for use in the Bahraini health system (Duron et al., 2016).

Operationalization of all relevant variables on the registry form were discussed with the data collection teams prior to implementation, and multiple editions of the data collection tool were created and refined to ensure local appropriateness and ease of use. For example, ED physicians requested that gunshot wounds and animal bites be removed from the checkbox options for the description of the injury as these are incredibly rare in the locale, and that sports injuries be added for ease of use. To ensure standardization of the data captured, multiple training sessions were held with the ED staff of each participating trauma center prior to starting data collection.Information on vitals included pulse, respiratory rate (RR), blood pressure, oxygen saturation (SaO2) and temperature. Injury severity at triage was studied using Glasgow Coma Scale (GCS). Information on the external location where the injury occurred was captured by the variable ‘Location of Injury’. The ‘Description of Injury’ variable consists of type and cause of injury.

To enable comparison of vitals across age categories, the values were first classified into normal/ abnormal using age-specific cut-offs for Pulse, RR, SaO2, systolic blood pressure (SBP) and temperature. The three age categories used for vitals interpretation were infant (birth to <1 year), young child (1-5 years), and older child (>5 years). For heart rate, the normal ranges were as follows: Infant (160-110bpm), young child (150-95bpm), and older child (120-80bpm). Normal ranges for respiratory rate were as follows: infant (40-30), young child (35-25), and older child (25-20). Normal ranges for systolic blood pressure were as follows: infant (90-80mmHg), young child (100-85mmHg), older child (110-90mmHg).

Any patient in the study with a SaO2 less than 94% was labeled to have an abnormal SaO2, and any patient in the study with a temperature outside of the range 36.5-37.5°C was labeled as having an abnormal temperature. These ranges follow the standards set by Lissauer et al (Lissauer, 2017). The proportions of patients who were found to have abnormal values of these variables for their age, are reported in the results.The age-specific pediatric trauma score (ASPTS) is a derived variable which captures trauma severity for different age-groups. Computation of ASPTS was done using a 2-step process. The first step involved comparing each patient’s GCS, SBP, pulse and RR to age-specific thresholds as defined by Potoka et al. and assigning a score to these individual variables (Potoka et al., 2001). In the second step, the sum of GCS, SBP, pulse and RR scores was taken to arrive at the ASPTS score for each patient.

The type and cause of injury are not reported separately in the medical records; therefore, this variable has been reported as it was captured at the source. The ‘Apparent intent’ variable captures information on whether the injury was a result of intentional harm or not. Whether or not an ambulance was used is captured by the variable ‘Ambulance Utilization’.

Diagnostic methods, interventions in the ED, and outcome (whether hospitalized or discharged) have been described using the variables: Imaging Done, Positive Trauma Findings Amongst Those with Imaging, ED Interventions and Hospital Disposition. Amongst ED interventions, systemic analgesia included any analgesia used on the World Health Organization (WHO) pain control ladder, (i.e. nonsteroidal anti-inflammatory drugs, morphine, etc.) (Yang et al., 2020).

**Fig. 1:**
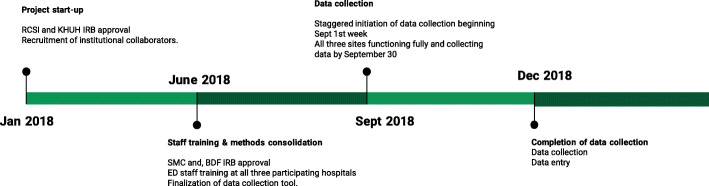
Timeline of PTR Implementation

Forms were completed exclusively by the physicians who rendered care to the patients in the ED upon initial presentation. The duration of data collection was three months (91 days in total), commencing on September 20th, 2018, and concluding on December 20^th^, 2018 (Figure 1). Registry forms were collected daily and then subsequently entered into a computerized database and double entered to assure accuracy.

### Statistical analysis

Categorical variables have been described as frequencies and column percentages. Data on characteristics of the patients have been stratified on age categories given the physiological differences between patients in these categories. Differences between characteristics of trauma patients and age categories was assessed using chi-squared test for categorical variables.

All analyses were performed using STATA version 16.1.

## Results

### Characteristics of pediatric trauma patients upon admission

Results shown are stratified by age categories because of the physiological differences between children of these age categories. Table 1 outlines the patient socio-demographic information and characteristics upon admission. The majority of the sample was composed of Bahrain Nationals (1128/1328, 84.9%), with the remainder of the sample being composed by Western Asia, Asia Pacific, North Africa, Gulf Cooperation Council (GCC), European Union (EU)/ United States of America (USA), or unknown nationalities.

**Table 1:** Demographics (*N* = 1328)

Variable	Number of Patients	Percentage of Patients
**Male**	880	66.3
**Female**	448	33.7
**<1 year**	23	1.73
**1 to 4 years**	454	34.18
**5 to 9 years**	450	33.88
**10–14 years**	401	30.19
**Bahrain**	1128	84.94
**Asia Pacific**	56	4.22
**EU**^**a**^**/USA**^**b**^	9	0.68
**North Africa**	22	1.66
**Other GCC**^**c**^	12	0.9
**Unknown**	25	1.88
**West Asia**	76	5.72

^a^European Union; ^b^United States of America; ^c^Gulf Cooperation Council

The severity of traumatic brain injury in terms of GCS categories was mild for most children (Table 2), although 3 patients (0.7%) in the age category of 1 to 4 years had very severe injury, as did 2 children (0.6%) in the age category of 10-14 years. For most younger children, the injury took place at home (60.9% in <1-year old’s, 76% in 1 to 4 year old’s and 48.2% in 5 to 9 year old’s), while school was the most common location (39.1%) where injuries took place for children in the oldest age category. Injuries occurring on the road were also notably high in all age categories. The most common description of injury was fall injuries (750 out of 1328, 56.48%) and the relative proportion of children with this injury type decreased with increasing age categories, with the highest proportion being in the youngest age category (73.9%) and lowest being in the oldest age category (50.4%). The majority of injuries were accidents. In the older age groups 0.5% of injuries were the result of intentional self-harm, and self-harm was highest in the 1-4 age group, accounting for 1% of injuries. The proportion of abnormal vitals was highest in infants - 41.7% of them presented with abnormal pulse, abnormal respiratory rate was found in 87.5% of infants, abnormal systolic blood pressure in 77.8%, abnormal oxygen saturation in 8.3%, and 11.1% of the infants presented with fever. Older children had significantly lower occurrence of abnormal vitals compared to infants.Vitals, GCS, apparent intent, ambulance utilization, and hospital disposition are the only variables with a small portion of missing data in Tables 2 and 3, this is attributable to loss of capture when the registry forms were filled out by the ED physicians.

**Table 2:** Characteristics of Pediatric Trauma Patients Upon Admission (*N* = 1328) ^a,b^

Variable [n,%]	<1 year (***n*** = 23)	1 to 4 years (***n*** = 454)	5 to 9 years (***n*** = 450)	10–14 years (***n*** = 401)	*p* value
**Sex (*n*** **= 1328)**					
Male [*n* = 880; 66.3%]	16 (69.6%)	285 (62.8%)	297 (66%)	282 (70.3%)	0.135
Female [*n* = 448; 33.7%]	7 (30.4%)	169 (37.2%)	153 (34%)	119 (29.7%)	
**Nationality (*n =*** **1328)**					
Bahraini (*n* = 1128; 84.9%)	20 (87%)	389 (85.7%)	386 (85.8%)	333 (83%)	0.649
Non-Bahraini (*n* = 200; 15.0%)	3 (13%)	65 (14.3%)	64 (14.2%)	68 (17%)	
**Glasgow Coma Scale Category (*n*** **= 1169)**					
Mild [13 to 15] (*n* = 1160; 99.23%)	17 (100%)	401 (98.9%)	401 (99.8%)	341 (99.1%)	0.838
Moderate [9 to 12] (*n =* 2; 0.17%)	0 (0%)	1 (0.2%)	0 (0%)	1 (0.3%)	
Severe [6 to 8] (*n =* 2; 0.17%)	0 (0%)	1 (0.2%)	1 (0.2%)	0 (0%)	
Very Severe [3 to 5] (*n* = 5; 0.42%)	0 (0%)	3 (0.7%)	0 (0%)	2 (0.6%)	
**Location of Injury (*n =*** **1328)**					
Home (*n* = 683; 51.42%)	14 (60.9%)	345 (76.0%)	217 (48.2%)	107 (26.7%)	< 0.0001
School (*n* = 236; 17.77%)	0 (0%)	2 (0.4%)	77 (17.1%)	157 (39.1%)	
Road (*n* = 137; 10.31%)	4 (17.4%)	29 (6.4%)	52 (11.6%)	52 (13.0%)	
Other (*n* = 272; 20.48%)	5 (21.7%)	78 (17.2%)	104 (23.1%)	85 (21.2%)	
**Description of Injury (*n =*** **1328)**					
Fall Injury (*n* = 750; 56.48%)	17 (73.9%)	289 (63.0%)	245 (54.4%)	202 (50.4%)	< 0.0001
Blunt Trauma (*n* = 153; 11.52%)	0 (0%)	34 (7.5%)	63 (14.0%)	56 (14.0%)	
Motor Vehicle Collisions (MVC) (*n* = 79; 5.95%)	2 (8.7%)	18 (4.0%)	29 (6.4%)	30 (7.5%)	
Sports Injury (*n* = 76; 5.72%)	1 (4.3%)	2 (0.4%)	16 (3.6%)	57 (14.2%)	
Crush Injury (*n* = 67; 5.05%)	0 (0%)	32 (7.0%)	17 (3.8%)	18 (4.5%)	
Eye Injury (*n* = 48; 3.61%)	0 (0%)	7 (1.5%)	27 (6.0%)	14 (3.5%)	
Foreign Body (*n* = 48; 3.61%)	1 (4.3%)	17 (3.7%)	21 (4.7%)	9 (2.2%)	
Laceration (*n =* 44, 3.31%)	0 (0%)	16 (3.5%)	19 (4.2%)	9 (2.4%)	
Burn (*n* = 36; 2.71%)	2 (8.7%)	22 (4.8%)	9 (2.0%)	3 (0.7%)	
Ingestion Injury (*n* = 21; 1.58%)	0 (0%)	15 (3.3%)	3 (0.7%)	3 (0.7%)	
Drowning/Near-Drowning (*n =* 6; 0.45%)	0 (0%)	5 (1.1%)	1 (0.2%)	0 (0%)	
**Apparent Intent (*n*** **= 1202)**					
Accident (*n* = 1153; 95.92%)	18 (94.7%)	405 (98.1%)	383 (95.0%)	347 (94.5%)	0.054
Assault (*n* = 41; 3.41%)	1 (5.3%)	4 (1.0%)	18 (4.5%)	18 (5.0%)	
Intentional Self Harm (*n =* 8; 0.67%)	0 (0%)	4 (1.0%)	2 (0.5%)	2 (0.5%)	
**Ambulance Utilization (*n*** **= 1248)**					
Yes (*n* = 47; 3.77%)	2 (10.5%)	15 (3.5%)	12 (2.8%)	18 (4.8%)	0.2
No (*n* = 1201; 96.23%)	17 (89.5%)	410 (96.5%)	416 (97.2%)	358 (95.2%)	
**Age-Specific Pediatric Trauma Score (ASPTS)**					
ASPTS< 10 (*n* = 25; 3.5%)	2 (28.6%)	7 (3.0%)	10 (4.4%)	6 (2.5%)	0.002
ASPTS> 10 (*n =* 683; 96.5%)	5 (71.4%)	229 (97.0%)	217 (95.6%)	232 (97.5%)	
**Pulse (*n*** **= 884)**					
Abnormal (*n* = 159)	5 (41.7%)	52 (16.7%)	35 (12.5%)	67 (24%)	0.001
Normal (*n* = 725)	7 (58.3%)	260 (83.3%)	246 (87.5%)	212 (76%)	
**Respiratory Rate (*n*** **= 801)**					
Abnormal (*n* = 464)	7 (87.5%)	176 (62.6%)	127 (49.6%)	154 (60.2%)	0.004
Normal (*n* = 337)	1 (12.5)%	105 (37.4%)	129 (50.4%)	102 (39.8%)	
**SaO2 (*n*** **= 868)**					
Abnormal	1 (8.3%)	4 (1.3%)	2 (0.7%)	0 (0%)	0.008
Normal	11 (91.7%)	299 (98.7%)	281 (99.3%)	270 (100%)	
**Systolic Blood Pressure (*n*** **= 783)**					
Abnormal (*n* = 360)	7 (77.8%)	130 (50.8%)	84 (32.4%)	139 (52.8%)	< 0.001
Normal (*n* = 423)	2 (22.2%)	126 (49.2%)	171 (67.1%)	124 (47.2%)	
**Febrile (*n*** **= 892)**					
Y es (*n =* 6)	1 (11.1%)	1 (0.3%)	1 (0.3%)	3 (1.1%)	< 0.001
No (*n* = 886)	8 (88.9%)	317 (99.7%)	282 (99.7%)	279 (98.9)	

^**a**^values are frequencies and column percentages for categorical variables.

^**b**^*p* values reported are for Chi-square for categorical variables..

**Table 3:** Hospital Interventions & Outcomes (*N* = 1328) ^a,b,c^

Variable	<1 year	1 to 4 years	5 to 9 years	10–14 years	*p* value
	(***n =*** 23)	(***n*** = 454)	(***n*** = 450)	(***n =*** 401)	
**Imaging Performed (*n =*** **1328)**					
Yes (*n* = 707; 53.23%)	12 (52.2%)	178 (39.2%)	215 (47.8%)	302 (75.3%)	
No (*n =* 621; 46.76%)	11 (47.8%)	276 (60.8%)	235 (52.2%)	99 (24.7%)	< 0.001
**Positive Trauma Findings Amongst Those with Imaging (*n =*** **707)**					
Yes (*n =* 261; 36.91%)	5 (41.7%)	60 (33.7%)	78 (36.3%)	118 (39.1%)	
No /Unknown (*n* = 446; 63.08)	7 (58.3%)	118 (66.3%)	137 (63.7%)	184 (60.9%)	0.672
**Emergency Department Interventions (*n*** **= 666; 50.15%)**					
Advanced Cardiac Life Support Trauma Resuscitation (*n* = 6; 0.90%)	1 (25.0%)	3 (1.3%)	1 (0.4%)	1 (0.5%)	< 0.0001
Fracture Management (*n* = 319; 47.9%)	1 (25.00%)	70 (31.0%)	90 (39.3%)	158 (76.3%)	
Wound Management (*n* = 303; 45.50%)	2 (50.0%)	139 (61.5%)	119 (52.0%)	43 (20.8%)	
Fracture + Wound Management (*n =* 5; 0.75%)	0 (0%)	2 (0.9%)	1 (0.4%)	2 (1.0%)	
Activated Charcoal (*n =* 1; 0.15%)	0 (0%)	1 (0.4%)	0 (0%)	0 (0%)	
Child Protective Services (*n =* 7; 1.05%)	0 (0%)	7 (3.1%)	0 (0%)	0 (0%)	
Antibiotics (*n =* 25; 3.75%)	0 (0%)	4 (1.8%)	18 (7.9%)	3 (1.4%)	
Systemic Analgesia (*n* = 649; 48.87%)	4 (57.1%)	183 (64.2%)	207 (71.4%)	255 (89.8%)	
**Hospital Disposition (*n*** **= 1297)**					
Hospital Admission (*n* = 19)	0 (0%)	11 (2.4%)	5 (1.1%)	3 (0.7%)	*p* < 0.0001
Admission to Surgery (*n* = 82)	6 (27.3%)	23 (5.1%)	32 (7.3%)	21 (5.2%)	
Mortality (*n =* 6)	1 (4.5%)	4 (0.9%)	0 (0%)	1 (0.2%)	
Treated and Discharged or Referred to Specialist (*n* = 1190)	15 (68.2%)	407 (91.5%)	404 (91.6%)	364 (93.6%)	

^**a**^values are frequencies and column percentages for categorical variables.

^**b**^*p* values reported are for Chi-square for categorical variables.

^c^ED intervention categories are not mutually exclusive and percentages may not add up to 100%..

### Diagnosis, treatment, and hospital admission

Diagnostic imaging was performed on 707 (53.2%) of the patients in the study, with 261 (36.91%) of these patients having positive trauma related findings on at least one imaging modality performed (i.e. X-Ray, CT Scan, or sonography for trauma). The likelihood of positive trauma findings upon imaging was not significantly different by age category.

Administration of systemic analgesia was positively correlated with age, with 89.8% of patients in the 10-14 age category receiving analgesia within the ED. Fracture management was the most common intervention in the 10-14 age group (158 [76.3%]), while wound management was the most common ED intervention performed on all other age categories. Antibiotic prescription was infrequent in the sample, however the 5-9 age category had significantly elevated incidence of receiving antibiotics. This correlates with the elevated incidence of receiving wound care in this age group.

Diagnostic imaging and ED interventions were not performed on all patients in the study, and only those who were documented as having received imaging or interventions were reported in Table 3. Amongst the patients in the study, 50.15% received some form of intervention in the ED. The remaining 49.85% of patients either were discharged from the ED without having received any interventions, or they were admitted to another department in the hospital where their care was managed. Regarding invasive ED interventions, six patients received advanced cardiac life support (ACLS) trauma resuscitation after presenting to the ED in traumatic cardiac arrest, and none of these patients survived the event.

Admission to the hospital ward was uncommon, with only 19 patients being admitted to medicine, however 82 patients were admitted for surgery. The <1 age category was significantly more likely to be admitted for surgery (26.1%) than any other age category. Also of note, is that all 7 of the children placed under the care of child protective services were in the 1-4 year old age category. The majority of the patients in the study were treated and discharged from the ED (69%).

### Motor vehicle collisions

MVC included any injury involving a motorized vehicle, including both passengers in vehicles, and pedestrians struck by vehicles. The risk of suffering an MVC was positively correlated with age, with 38.5% of patients in the 10–14 age category and 2.6% of patients in the > 1 age category (Table 4).

**Table 4:** Characteristics of Road Traffic Injury Patients (*N* = 78) ^**a,b**^

Variable	Pedestrian Struck (***n*** = 37)	Vehicle Collision (***n*** = 41)	Total RTA (***n*** = 78)	***p*** value
**Sex**				
Male	31 (83.8%)	18 (43.9%)	49 (62.8%)	< 0.001
Female	6 (16.2%)	23 (56.1%)	29 (37.2%)	
**Age**				
< 1	0 (0%)	2 (4.9%)	2 (2.6%)	0.002
1 to 4 years	3 (8.1%)	15 (36.6%)	18 (23.1%)	
5 to 9 years	20 (54.0%)	8 (19.5%)	28 (35.9%)	
10–14 years	14 (37.8%)	16 (39.0%)	30 (38.5%)	
**Nationality**				
Bahraini	25 (67.6%)	33 (80.5%)	58 (74.4%)	0.192
Non-Bahraini	12 (32.4%)	8 (19.5%)	20 (25.6%)	
**Hospital**				
Salmaniya Medical Complex	14 (37.8%)	11 (26.8%)	25 (32%)	0.582
Bahrain Defense Force Hospital	20 (54%)	26 (63.4%)	46 (59%)	
King Hamad University Hospital	3 (8.1%)	4 (9.8%)	7 (9%)	
**Ambulance utilization (*n*** **= 74)**				
Yes	7 (20%)	14 (35.9%)	21 (28.4%)	0.13
No	28 (80%)	25 (64.1%)	53 (71.6%)	
**Glasgow Coma Scale Category (*n =*** **70)**				
Mild (13 to 15)	32 (94.2%)	35 (97.2%)	67 (95.7%)	0.583
Moderate (9 to 12)	1 (2.9%)	0 (0%)	1 (1.4%)	
Severe (6 to 8)	0 (0%)	0 (0%)	0 (%)	
Very Severe (3 to 5)	1 (2.9%)	1 (2.8%)	2 (2.9%)	
**Pulse (*n*** **= 57)**				
Abnormal	5 (19.2%)	6 (19.3%)	11 (19.3)	0.991
Normal	21 (80.7%)	25 (80.6%)	46 (80.7%)	
**Respiratory Rate (*n =*** **48)**				
Abnormal	9 (40.9%)	13 (50%)	22 (45.8%)	0.529
Normal	13 (59.1%)	13 (50%)	26 (54.2%)	
**SaO2 (*n*** **= 55)**				
Abnormal	0 (0%)	0 (0%)	0 (0%)	
Normal	24 (100%)	31 (100%)	55 (100%)	
**Systolic Blood Pressure (*n =*** **55)**				
Abnormal	12 (48%)	19 (63.3%)	31 (56.4%)	0.254
Normal	13 (52%)	11 (36.7%)	24 (43.6%)	
**Febrile (*n*** **= 56)**				
Yes	0 (0%)	1 (3.1%)	1 (1.8%)	0.382
No	24 (100%)	31 (96.9%)	55 (98.2%)	
**Age Specific Pediatric Trauma Score (ASPTS) (*n =*** **48)**				
**ASPTS < 10**	1 (4.3%)	0 (0.0%)	1 (2.1)	0.292
**Imaging done**				
yes	28 (75.7%)	18 (43.9%)	46 (59%)	0.004
No	9 (24.3%)	23 (56.1%)	32 (41%)	
**Trauma Related Imaging findings (*n =*** **261)**				
Yes	9 (32.1%)	4 (22.2%)	13 (28.3%)	0.466
No / Unknown	19 (67.9%)	14 (77.8%)	33 (71.7%)	
**ED Interventions (*n =*** **36)**				
ACLS/Trauma Resuscitation	2 (10.5%)	2 (11.8%)	4 (11.1%)	0.823
Fracture / Wound Management	5 (26.3%)	3 (17.6%)	8 (22.2%)	
Systemic Analgesia	12 (63.2%)	12 (70.6%)	24 (66.7%)	
**Hospital Disposition (*n =*** **19)**				
Hospital Admission	2 (5.7%)	2 (5.1%)	4 (5.4%)	0.911
Admission to Surgery	7 (20.0%)	5 (12.8%)	12 (16.2%)	0.403
Mortality	1 (2.7%)	2 (4.9%)	3 (3.8%)	0.618

^**a**^values are frequencies and column percentages for categorical variables.

^**b**^*p* values reported are for Chi-square for categorical variables.

Pedestrians struck were more likely to be males than females (83.6% vs. 16.2%, respectively). However, females were more likely to be a passenger in a vehicle collision (56.1%) when compared with males (43.9%). Overall, male children were more likely to suffer an MVC (62.8%). MVC data was collected to include motorcyclist and pedestrians struck by motorcyclists, however no such events were captured during the study period. Cycling collisions that involved non-motorized bicycles were reported as fall injuries.

In terms of ED interventions, systemic analgesia was administered to a third of all MVC patients. There was no statistically significant difference in systemic analgesia administration for different age categories of patients who suffered an MVC. Imaging was done on 59% of MVC patients and positive trauma related imaging findings were found in 28.3% of patients. Patients involved in vehicle collisions were more likely to arrive at the ED via ambulance when compared with pedestrians who were struck by a vehicle (35.9% vs 20% respectively).

BDF hospital received the most MVC patients in both the pedestrian struck (54%) and vehicle collision (63.4%) subcategories when compared with the other two hospitals involved in receiving pediatric trauma. The majority (95.7%) of patients who suffered a MVC presented to the ED with a mild GCS score (13–15). However, two patients presented with signs of severe brain injury (GCS of 3–5). Both these patients were pronounced dead in the ED.

For patients who were involved in vehicle collisions, it was noted that 20.5% were front seat passengers. Of the 41 patients involved in vehicle collisions, 31 were seated in the back seat (79.5%), 8 were in the front seat (20.5%), one child under the age of 10 was seated in the front seat (2.5%), and one child’s seating was not recorded (2.5%). In Bahrain, the minimum legal age for a front seat passenger is 10 years. A third of the vehicle collisions occurred at high speed (> 40 km/h) and 15.4% of collisions had airbag deployment. Furthermore, seat belt utilization was reported to be extremely low (7.7%) with ejection from the vehicle cabin occurring in 12.8% of passengers.

Four patients were admitted to the hospital and 12 were admitted to surgery amongst those in MVC. Six death pronouncements were made in the ED due to trauma during this pilot study, half of which were due to MVCs. One occurred due to a pedestrian being struck. The other two were passengers in vehicles, one of which suffered ejection from the vehicle cabin.

## Discussion

A total of 1328 patients were included in the study. Sixty-nine percent of patients were treated and discharged from the ED, 30.5% were admitted to the hospital, admitted for surgery, or seen by a specialist, and 0.5% were pronounced deceased. The percentage of patients documented as unrestrained during Motor Vehicle Collisions (MVC) was 92.3%, and amongst those involved in MVC, 12% were ejected from the cabin of the vehicle. These alarming results provide a description of the distribution of pediatric injury in trauma centers in Bahrain, which was the primary aim of the study.

### Demographics and hospital presentation

There was a notable split in the ratio between males and females in the study, with a 2:1 male to female ratio being demonstrated. When looking at patient characteristics, similar distributions amongst sexes have been observed in trauma studies around the GCC region. One study conducted by Alyafei et al outlined that in a pediatric trauma registry in Qatar, 82.8% of the injuries were amongst male patients (11) Additionally, a study conducted by Alghnam et al found that 85% of the MVC injuries in Saudi Arabian general population were in males (Alghnam et al., 2014). These findings are in line with the results of this study, where 66.3% of the study sample is male.

While the general population of Bahrain is composed significantly by non-nationals, this is not reflected in the pediatric population as the majority of non-bahrainis are migrant workers who are above legal working age and do not add to the pediatric population. This may help to explain why Bahraini children represent the majority of the patients presenting to the ED in the study.

Most pediatric patients arrived at the ED by private vehicle, with only 3.76% being brought to the ED by ambulance. In the United States, it was estimated that 8% of all-comers in the pediatric ED were brought in by ambulance, with the significant majority ofthose patients presenting for traumatic injury (Augustine, 2014; Shah et al., 2008). Ambulance use for pediatric patients in the study sample was significantly lower than other high-income countries (Shah et al., 2008). EMS in the Kingdom of Bahrain has since been revitalized with the official launch of the National Ambulance Project in June 2019, however none of the patients in our study had access to this service, as data collection concluded in December of 2018 (Abuzeyad et al., 2020).

Repeat visitation was investigated, as “Hospital shopping” was raised as a concern by local ED staff during registry implementation. None of the patients in the study presented to multiple hospital EDs within 24 hours of an injury. 15 individuals were identified as having presented to the ED twice during the study, however these were all separate injuries and treated as different events in the registry.

Some of the variables for vitals had missing data at the source of the data collection. This is most likely due to a delay in measurement of these variables due to clinical priorities at the time of triage, and is less likely to be reflective of an inherent issue with the trauma registry itself. 

ASPTS <10 has been used previously as an indicator of severe trauma presentation, and has been documented to be a strong predictor of mortality. The number of patients in the dataset with ASPTS<10 was low (25 [1.88%]). ASPTS was found to be <10 significantly more frequently in the <1 age category (28.6%) demonstrating higher trauma severity amongst patients in that age category. This can also be explained by the increased frequency of abnormal vital signs in the age category.

### Description of injury

Analysis has demonstrated that outcomes of pediatric traumatic injuries have a gender and injury-specific pattern in Bahrain. This discrepancy is particularly evident when considering MVCs. Male children were found to have significantly higher odds of being struck as pedestrians when compared with females in the study sample (odds ratio: 6.6 [95% CI: 2.0–23.1]; *p-*value = 0.000).

Albeit infrequent, MVCs put children in need of emergent surgical management and prolonged hospital care (15% of surgical admissions occurred due to MVCs). Bahrainis had significantly lower odds of being in an MVC compared to non-Bahrainis (odds ratio: 0.49 [95% CI: 0.28–0.88]; *p-*value = 0.0071), who were almost twice as likely to be in an MVC.

MVCs were identified as one of the most common (50%) description of injury leading to pediatric mortality. Furthermore, it was found that seat belt utilization was extremely low, with a very high portion of the sample population involved in MVC being unrestrained during the collision (92.3%).

Previous estimates of seatbelt utilization in the GCC already are significantly lower than those of other high income countries, however these findings are much lower than even those estimates (Abbas et al., 2011). Previous studies of MVC prevention have demonstrated that a three-pronged approach including enforcement of seat belt laws, strengthening of the traffic court system, and awareness campaigns for MVC prevention can be effective at increasing seat belt usage. Adoption of similar public health initiatives in Bahrain may help to protect the pediatric population from avoidable MVC fatalities (Amiotte et al., 2016).

Drownings and near drownings were rare events, however they caused half of the mortality and significant morbidity in the study population. Six patients experienced drowning/near-drowning;

half of them were pronounced deceased in the ED, two were admitted to the pediatric intensive care unit (PICU), and one was admitted to the pediatric ward. Given the poor outcomes associated with these types of events, robust prevention practices can have a dramatic impact in reducing the incidence of drownings. Effective evidence based pediatric drowning prevention centers on constant adult supervision, pool isolation fencing, personal flotation devices, and swimming education for children over the age of four; these interventions could be implemented in Bahrain in an attempt to prevent the mortality and morbidity attributable to drownings (Conover & Romero, 2018).

Fall injuries accounted for the majority of the description of injuries for patients presenting to the ED (56.48%), and was negatively correlated with increasing age, which is typically expected given developmental norms. However, the portion of ED presentations attributed to falls is concerningly higher than what was found in previous articles studying the same age categories (Aoki et al., 2019; Nesje et al., 2019).

While falls accounted for the majority of injuries across age categories, sports injuries were the second leading description of injuries for the oldest age group (14.2%). This likely coincides with the introduction of sporting events and intramural athletics in this age category.

The vast majority of injuries documented in this study were accidental (95.52%), and occurred in the home (51.42%). However, seven patients in the study were placed under the care of child protective services. Based on previous studies in Bahrain, only 10% of child abuse victims are placed in child protective services, thus it is possible that this figure does not fully capture the extent of injuries caused by physical child abuse (9)(Bahrain, 2012). Only 3.4% of injuries in the study were reported as non-accidental injury.

Burn injuries were not common (n=36), but have been previously associated with non-accidental injuries despite all of the burn injuries in this study having been reported as accidental (9). One patient of the 36 who sustained burned injuries was placed under child protective services.

### Limitations

This trauma registry is the first such initiative in the country of Bahrain, however, there are certain limitations in the present body of work which can pave the way for further large-scale research. First and foremost, data was captured exclusively for patients presenting to the ED at major trauma centers. All minor trauma that was not deemed severe enough to warrant presentation to an ED, or which presented to a local clinic for care, was not captured in this study.

Using a paper-based data collection tool that was completed by the ED physician placed forms at risk of physical damage to the paper forms and poor capture of data due to being misplaced. Because data was collected for only three months, the study was unable to investigate the impact of seasonality on pediatric trauma in Bahrain. Additionally, the participating hospitals in this study were all government/military hospitals and we could not capture the trauma trends in minor trauma cases that present to exclusively private hospitals.

Given the method of data collection in the ED, it is possible that some of the injuries that were categorized as falls may have actually occurred on a bicycle or other non-motorized method of transportation. This would have been documented based upon the discretion of the triaging physician at the point of data collection. Subsequently this may have caused a reduction in the number of injuries recorded as MVC. 

While the creation of a pilot trauma registry was successful and gave a glimpse into what implementing a sustainable registry might involve, a systematic approach to explore barriers to registry sustainability was not able to be performed, and therefore only informal reporting from hospital administrators and study coordinators was collected.

### Registry sustainability

Several obstacles to the implementation of a sustainable registry were uncovered over the course of the study. The most glaring, is the necessity for the national registry to be incorporated into electronic medical records. Paper based registry forms demanded a tremendous amount of effort and resources which were taxing both on the ED staff as well as the research team. Efforts to streamline data collection into the patient charts should be made a priority, as it would be the most robust and efficient method of surveillance. The current move towards electronic medical records in the country makes implementation of a comprehensive national trauma registry well poised to be incorporated into current efforts.

Investment of funding into the creation of designated support staff to create and maintain a trauma surveillance system would additionally be a crucial next step for injury surveillance in Bahrain. The addition of this workload onto existing hospital staff would not be feasible in the current system. In addition, trauma data is currently siloed independently, both by institutions and by prehospital vs. in-hospital record systems. A sustainable national injury surveillance system would need to collect and aggregate the data from all these institutions and have the respective institutions invest by creating methods by which this data can be shared securely and without risk.

## Conclusion

There are significant implications that this study holds for policy implementation and practice surrounding injury prevention in the Kingdom of Bahrain. Having documented the low estimates of seat belt utilization amongst pediatric MVC, and subsequently how frequently pediatric MVC victims are ejected from the cabin of the vehicle, a call to action to implement immediate public health policies to protect children from the morbidity and mortality associated with MVC in the country is warranted.Similarly concerning is the high morbidity and mortality associated with drownings and

near-drownings in the country. Despite being rare, these injuries carried half the mortality in the study, and thus public health policy aimed at preventing drownings should be implemented.

The PTR in Bahrain was a step forward in understanding pediatric trauma, however more studies exploring the epidemiology of trauma in the Kingdom of Bahrain are necessary to guide specific targeted interventions, and to evaluate how prevention policies are impacting injury trends. A national injury surveillance system would be well poised to address the gaps in knowledge uncovered by the PTR study by enabling Bahrain to advance its trauma system, and serve to protect the children in Bahrain from suffering preventable injuries.

## Data Availability

The dataset used and analysed during the current study is available from the corresponding author upon reasonable request.
